# Alcohol and older people: A systematic review of barriers, facilitators and context of drinking in older people and implications for intervention design

**DOI:** 10.1371/journal.pone.0191189

**Published:** 2018-01-25

**Authors:** Sarah Kelly, Olawale Olanrewaju, Andy Cowan, Carol Brayne, Louise Lafortune

**Affiliations:** Cambridge Institute of Public Health, Forvie Site, University of Cambridge School of Clinical Medicine, Cambridge, United Kingdom; Nathan S Kline Institute, UNITED STATES

## Abstract

**Background:**

Harmful alcohol consumption in older people has increased and effective approaches to understanding and addressing this societal concern are needed.

**Methods:**

Systematic review of qualitative studies in older populations (55+ years) to identify barriers, facilitators or context of drinking in older people. Multiple databases (MEDLINE, EMBASE, PsycINFO, CINAHL, CENTRAL, Social Sciences Citation Index, York Centre for Reviews and Dissemination, Cochrane database and grey literature) were searched from 2000 to February 2017 for studies in English, from OECD countries using MeSH terms and text words relating to alcohol combined with older age terms. Study quality was assessed using NICE methodology. The review is reported according to PRISMA.

**Results:**

Drinking in older people is strongly linked to social engagement and there is scepticism about the health risks of alcohol. Drinking was also linked to difficulties such as social isolation, illness or bereavement. Alcohol can be related to routines and identity. However, older people often regulate their own drinking and strategies that emphasise the life experience of older people to drink wisely could be helpful.

**Conclusions:**

To be effective societal approaches need to take into account contexts of risks for harmful drinking. The evidence supports a strong social role for drinking alcohol which should be taken into account in any policy development with the potential benefits of social participation for cognitive health. Approaches to reducing alcohol use in older people need to avoid paradoxical harm, with a need for approaches that reduce harm from drinking alcohol but retain the benefit of socialising.

## Introduction

With an ageing population, it is increasingly important that older adults are supported to maintain and improve their physical, mental and social health and wellbeing, and to minimise risk of non-communicable diseases. Recent UK national guidelines and recommendations have linked alcohol consumption with a range of health conditions including heart disease, cancer, dementia and mortality as well as risk of accident and injury [1, 2].

Risky drinking is increasing in older people [3], and they may be at greater risk of alcohol-related harm because of physiological changes associated with ageing, chronic conditions or frailty, risk of falls or interactions with medication so that harmful effects of alcohol may manifest at lower levels of consumption [4]. However, older people are a heterogeneous population that also includes those experiencing ‘healthy ageing’ and consideration needs to be given to their different situations and circumstances. While many may have started drinking earlier in the lifecourse, about a third of older people drinking at harmful levels started consumption at these levels in later life [4].

In order to inform health promotion programmes and design interventions that are tailored to meet the needs of older people, an understanding of the context in which older people drink, and barriers and facilitators to changing behaviour in older age is important. To date there have been no systematic reviews of qualitative studies to provide this evidence. Qualitative studies can provide contextual evidence beyond that solely of effectiveness and ‘places the spotlight on making sense of behaviour and action in the context in which it occurs’ [5].

The work reported here was part of a comprehensive evidence synthesis relating to modifiable health behaviours in older people, preventive interventions and barriers, facilitators and context to inform policy and identify evidence gaps relating to ageing well and cognitive health conducted for the NIHR School of Public Health Research Ageing Well Programme.

The specific question addressed in this systematic review is:-

What issues (context, barriers and facilitators) prevent or limit, or help and motivate the prevention or reduction of excess alcohol consumption in people in older age (55+ years)?

## Methods

A systematic review of qualitative studies relating to alcohol or drinking behaviour in older populations. The protocol was pre-registered on PROSPERO [6]. The review is reported according to PRISMA [7].

### Search strategy and selection criteria

Search strategy: Multiple databases (MEDLINE, EMBASE, PsycINFO, CINAHL, CENTRAL, Social Sciences Citation Index, York Centre for Reviews and Dissemination, the Cochrane database, grey literature, including relevant websites) were searched from 2000 to February 2017 for systematic reviews or primary qualitative studies in English, from Organisation for Economic Co-operation and Development (OECD) countries using MeSH terms and text words relating to alcohol consumption and behaviour combined with older age terms (S1 File). A search filter was used to identify qualitative studies (S1 File). The searches were part of broader searches for a series of reviews covering a range of health behaviours, interventions, barriers and facilitators. Additionally, the reference lists of included studies and relevant reviews were searched.

Types of study and design: Primary qualitative studies or mixed methods studies with a qualitative component (using interviews, focus groups, ethnographic approaches or questionnaires with open ended responses), reporting barriers, facilitators or context of drinking in older people; or qualitative reports associated with specific interventions were eligible for inclusion. Studies focused on treatment of alcohol dependence were excluded.

Population: Older people aged 55 and over, living in the community; including healthy participants; with pre-conditions for later ill health such as high blood pressure, high cholesterol, overweight or obese, impaired cognitive function, functional limitations; on medication that did not affect outcomes; disadvantaged and minority groups. Studies primarily focused on populations with ill health e.g. stroke, coronary heart disease, mental health conditions were excluded.

Identification of relevant studies: Titles, abstracts and papers were screened for inclusion by two reviewers. Differences between reviewers’ results were resolved by discussion with a third reviewer. Studies excluded at the full paper screening stage are listed in S2 File along with the reason for exclusion.

### Quality assessment/risk of bias

Methodological quality was assessed using NICE methodology for qualitative studies [8] by one reviewer and checked for accuracy by a second reviewer. Differences between reviewers were resolved by discussion. No studies were excluded on the basis of quality.

### Data extraction and synthesis

Data relating to population and study characteristics of the included studies were extracted by one reviewer (SK) and checked by another reviewer (LLF, AC) (Table 1). Education or occupation details were sought as a proxy for socioeconomic status (SES) if specific SES data was not available.

**Table 1:** Characteristics of included studies.

Study	Country	Age (yrs)	Population, setting	Study objective
**Qualitative studies**				
Burrus 2015 [14]	US	Mean: 81.5 SD 7.5 Range 68–90	N = 11 older adults living independently in a congregate retirement community, who were regular drinkers; alcohol available at onsite pubs and shops; 45.5% male; 54.5% female.	Understanding older adults’ attitudes and beliefs about drinking: perspectives of residents in congregate living.
Dare 2014 [19]	Australia	65–74 Mean: 69.7 SD 3.3	N = 20 men and N = 22 women who were living in either private residences or (secular, resident-funded) retirement villages; 47.6% male; 52.4% female	To identify relationships between social engagement, setting and alcohol use.
Haarni 2010 [20]	Finland	60–75	N = 31 Urban older adults; 48.4% male; 51.6% female	To examine 60–75 year olds relationship to alcohol.
Haighton 2016 ^a^ [10]	UK (England)	50+ Range: 50–95	N = 24 (qualitative interviews) and N = 27 (focus groups) older adults recruited through Age UK and regional services for alcohol problems; 50% male; 50% female (interviews); 22.2% male; 77.8% female.	Experiences of and attitudes towards services providing support for alcohol related health issues in people aged 50 and over.
Johannessen 2015 [21]	Norway	65+ Mean: 81 Range: 65–92;	N = 16 older people that received in-home nursing service or home-help services (N = 14 were widows or widowers); 37.5% male; 62.5% female	Older peoples’ experience with and reflections on use and misuse of alcohol and psychotropic drugs.
Joseph 2012 [11]	Canada	Mean: 61 Range: 44–74	Older male cricket (non-league, friendly) players of Afro-Caribbean origin and spectators (male and female). N = 27 formal interviews plus data collected by observation, casual conversation; mainly male	To understand alcohol use in older Caribbean-Canadian men
Kim 2009 [13]	Canada	60+ Mean: 72 SD 5.9 Range: 62–83	N = 19 elderly Korean immigrants residing in Canada (14 men, 5 women); 73.7% male; 26.3% female	To explore drinking culture, alcohol and alcohol use in older Korean immigrants in Canada.
Millard 2008 [12]	UK (Scotland)	65+	N = 90 staff and managers providing home, day, and residential care to elderly clients; gender not reported	Alcohol and service gaps in homecare for older people.
Reczek 2016 [16]	US	1) Mean: 63.5 Range 40–87 2) Mean 59 Range 40–89	1) Both spouses in 21 long-term (>7 years) marriages (n = 42); and 2) men and women (N = 46) in first marriages, remarried, divorced, never married or widowed;.1) and 2) 50% male, 50% female	Relationships between marital history and alcohol use in older adults.
Tolvanen 2005 [22]	Finland	90+	N = 181 participants who mainly lived in their own homes though some were in service housing or in nursing homes; 33.5% male; 76.5% female.	Alcohol in life story interviews with Finnish people aged 90 or over.
Ward 2011 [15]	UK	Range: mid 50s to late 80s	N = 21 individual interviews and N = 3 focus groups. Diverse range of older people, including living in their own homes, sheltered housing, hostels; 61.9% male, 29.1% female (interviews)	Older people’s perspectives on alcohol use in later life.
Wilson 2013^a^ [9]	UK (England)	50+ Range: 50–95	N = 24 (qualitative interviews) and N = 27 (focus groups) older adults recruited through Age UK and regional services for alcohol problems; 50% male; 50% female (interviews); 22.2% male; 77.8% female.	To understand older people’s reasoning about drinking in later life and how this interacted with health concerns.
**Studies with a limited qualitative component**				
Aira 2008 [18]	Finland	75+ (75–84: 83.4% 85+: 16.6%)	N = 699 home-dwelling elderly living in the community; 30.5% male; 69.5% female.	To describe alcohol use as self-medication by people aged over 75 years.
Borok 2013 [17]	US	Mean: 68.7 SD 6.6	N = 399 older ‘at-risk’ drinkers (identified by screening) who had taken part in an RCT^b^ to reduce drinking; 69% male, 31% female.	To understand why older at-risk drinkers decide to increase, decrease or maintain alcohol consumption after participation in an RCT aimed at reducing drinking.

^a^ Different papers using data from the same participant sample

^b^ RCT = randomised controlled trial

To identify information relevant to barriers, facilitators and context of drinking in older people, one researcher (SK) examined the results and discussion sections of each text, line by line, to identify data relating to older people and drinking, and coded text using NVivo (Version 9). Interpretation and concepts of the study authors were also included if they were developed from the original data. The coded text was then further examined and re-organised into themes (Table 2). Further interpretation and analysis was then conducted to develop potential alcohol prevention or reduction strategies in older people from the themes identified from the texts.

**Table 2:** Barriers, facilitators and the context of alcohol consumption in older people.

Influences on drinking	Context, barriers, facilitators	References
Social life	Alcohol as a facilitator or as an integral part of socialisation	[9][10][11][13][15][17][19] [20][21][22]
	Alcohol as a treat/something special/special occasions	[9][10][13][14] [19] [20]
	Fun and enjoyment	[11][13][15][19][20][22]
	Quality of life	[9] [15]
Social environment	Social norms	[9] [19] [20]
	Childhood and lifecourse norms/continuity with earlier life	[9] [14] [15]
	Moralistic attitudes to drinking	[9] [15][22]
	Influence of drinking habits of spouse/partner/family members/peers	[10] [14] [15][16][20]
	Drinking as a habit or as part of a regular routine	[10] [14] [19]
	Retirement–could both increase or decrease drinking	[9] [14] [15] [19] [20]
Health issues	*Context and reasons for drinking*	
	Drinking for medicinal purposes/health benefits	[9][13][17][18][20][22]
	Drinking for relaxation	[9][13][14][15][19][20]
	Ill health as reason for increasing drinking (cancer)	[17]
	Heavy use acceptable if in good health	[9]
	*Context and reasons to limit drinking*	
	Alcohol in the context of ageing (not able to drink as much)	[9] [10] [14] [20] [21]
	Ill-health or taking medication	[9][15][17][20]
	Diets/weight loss	[10][17]
	To improve health or maintain health so they could travel and maintain longer relationships with grandchildren or great grandchildren.	[10]
	Fear of falling or appearing foolish	[10]
Drinking to deal with difficulties	Drinking to deal with difficulties e.g. social isolation, illness, loss of physical health or mobility, bereavement such as loss of partners, family or friends	[9] [10] [15] [19][20][21]
Health messages	Scepticism about health advice; mixed messages; not personally relevant; health messages perceived as preaching	[9] [15] [18] [19] [21] [22]
	Confusion about units	[10]
Access issues	Cost and availability	[13][14][15][20]
Identity	Positive versus negative alcohol identities; controlled versus uncontrolled drinking	[9][13][14][15][20][22]
	Maintaining routines and identity in the context of role loss and functional limitations; connection with earlier life	[9][14][15]
	Accumulated life experience to drink moderately	[20]
Self-regulating strategies	e.g. only drinking with a meal or waiting till after a certain time in the day to have a drink	[10][13][15][18][19][20]
Barriers to giving up drinking	Consequences of not drinking: loss of an enjoyable part of their lives; more difficult to enjoy socialising if did not drink	[10] [15]
Barriers to receiving help	Perceived GPs as not wanting to treat drinkers, or did not see drinking as a legitimate illness to trouble a doctor with, embarrassing to admit problems.	[10]
Sources of help	GPs seen as primary source of help	[10] [15]
	GP advice to cut down might motivate reduction in drinking	[10][15]
Driving	Limited consumption when driving	[19]
**Specific population subgroups**		
Gender	Male and female ‘roles’; In men, alcohol used to mask degeneration, declining ability. In one study [10] drinking patterns were not strongly defined by gender, although some gender roles in relation to drinking were described.	[9][11][13][15][16][20][22]
Cultural aspects	Identity in immigrant populations–relating to types of drink, socialisation.	[11] [13]
People receiving homecare services	Potential support role of home and day care services and workers; bulk orders for alcohol cheaper than ordering a small amount	[12]

## Results

**Fig 1:**
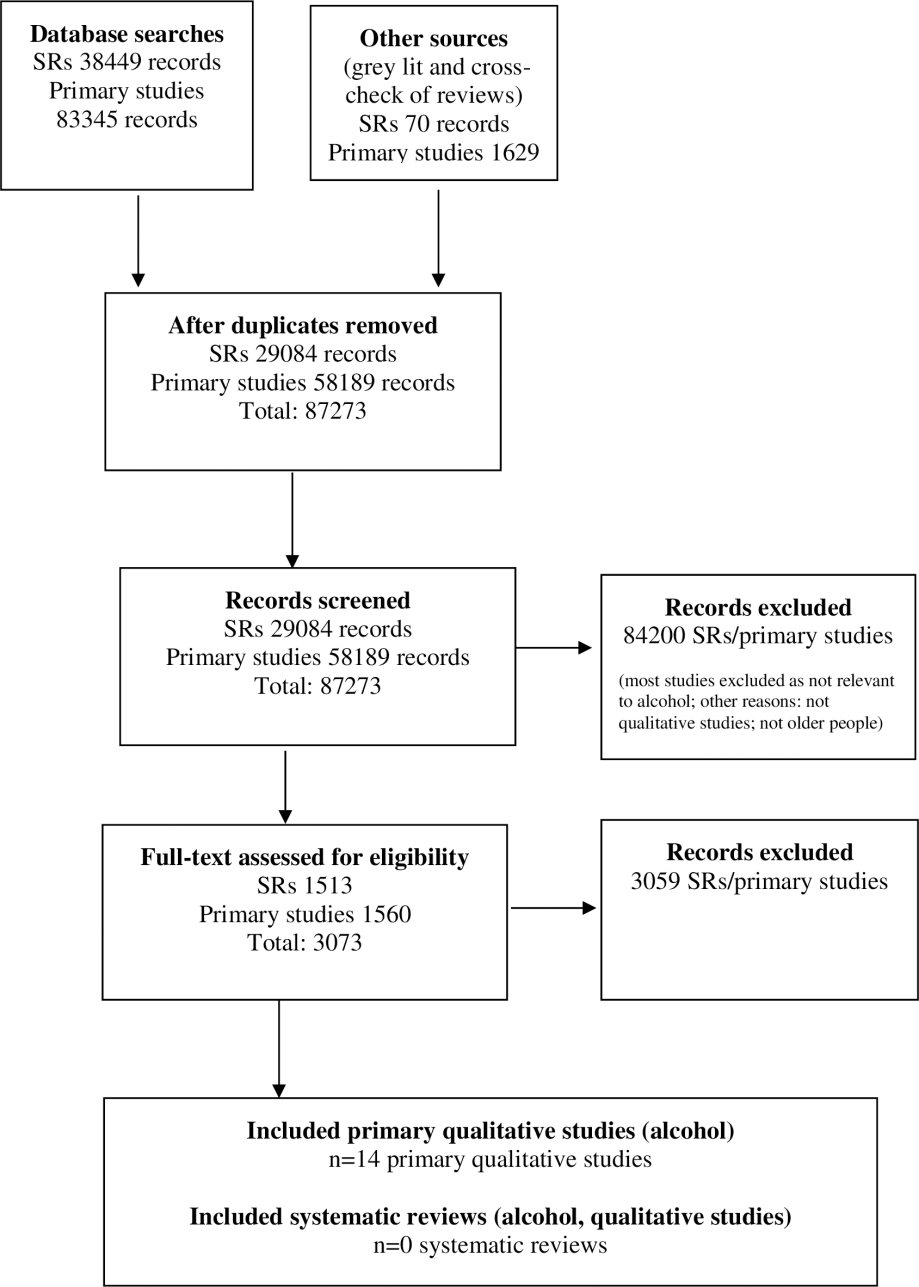
PRISMA flow diagram.

Fig 1 illustrates the flow chart for the study selection process. 14 qualitative papers were identified, of which 2 used data from the same study population [9, 10]. A summary of included studies, and the populations and context in which they were conducted is shown in Table 1 (further details in S3 File).

### Description of included studies

Four papers were from studies conducted in the UK, 3 in Finland, 3 in US, 2 in Canada, one in Norway and one in Australia. Twelve papers collected and reported full qualitative data—seven papers collected data through interviews, two from focus groups, two from a combination of interviews and focus groups and one used formal and informal interviews, casual conversation and observation. (S3 File). Two mixed methods studies collected and reported only very limited qualitative data using brief interviews.

Twelve studies included both male and female participants, one predominantly male participants [11], and one did not report gender [12]. Two studies were conducted in immigrant populations in Canada [12, 13], one study was in an all-Caucasian population [14], two included participants from both white and black and minority ethnic (BME) groups [15, 16] and one included predominantly white and Hispanic populations [17]. Ethnicity was not reported in other studies. Only 5 studies reported SES of participants or a proxy [16–20]. Of these one recruited from predominantly high socioeconomic areas [19], the other studies included a range of socioeconomic groups, none specifically recruited people from low SES groups (S3 File).

Details of alcohol consumption of study participants where available are shown in S3 File. Information about amount and distribution of drinking patterns of participants was limited in some studies. Two studies specifically stated inclusion of only current or regular drinkers [13, 14]. Two studies did not report details of alcohol consumption of participants [12, 16]. Most studies appeared to include participants drinking at a range of levels, though this was sometimes not clearly reported. A number of studies included some abstainers, including past drinkers [9, 10,18,20–22]; one study reported excluding lifetime abstainers [20]. Two papers using the same study participants clearly reported the distribution of drinking patterns of participants [9, 10] and included occasional minimal drinkers, moderate and heavy drinkers, currently abstinent previously dependent drinkers and a small number of dependent drinkers. Studies solely in dependent drinkers were not included, so where a small number of participants were dependent drinkers, the evidence extracted has focused on non-dependent drinkers where possible [9, 10]. Only one study was found, with limited qualitative data, which specifically included only ‘at risk’ participants [17].

#### Quality assessment

Quality assessment results and assessment criteria of individual studies is shown in S4 File. Overall, quality of studies was generally high or moderate, with the qualitative component of only 3 studies rated as low [12, 17, 18].

### Barriers, facilitators and context of drinking in older people

**Fig 2:**
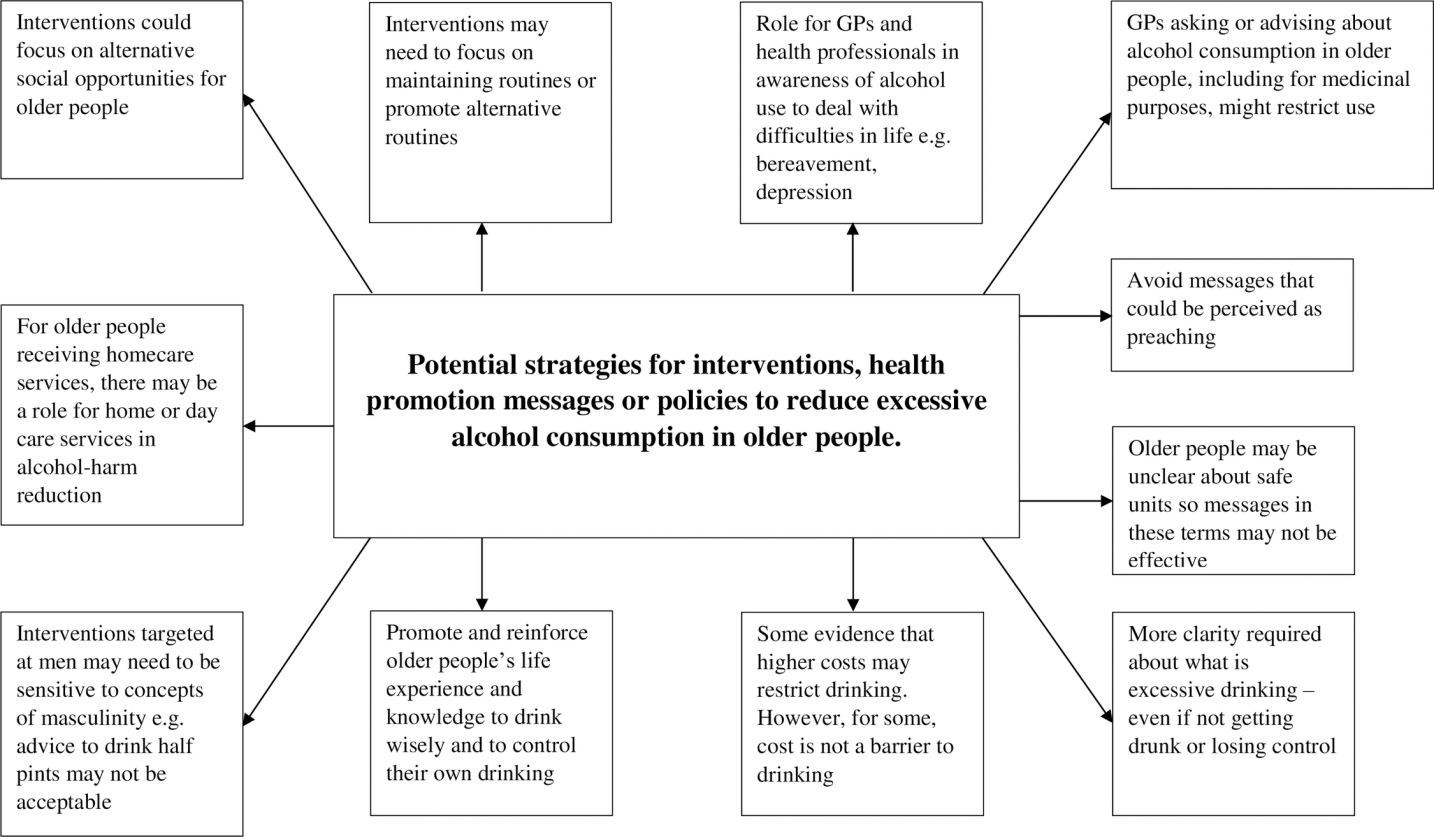
Potential strategies to reduce alcohol consumption in older people.

Few studies specifically identified topics as barriers or facilitators, most of the findings related to the context in which older people drink, attitudes to drinking, and drinking in the context of ageing. While studies had differing objectives, populations and context, some themes were consistently repeated across several studies (Table 2). Of the main themes described below, none were solely identified in studies rated as lower quality–these issues were also raised in other higher quality studies. Potential alcohol prevention or reduction strategies in older people developed from this evidence are shown in Fig 2.

#### Alcohol as part of social life, fun and enjoyment

Alcohol use was linked with social life and enhancing social engagement in almost all studies (Table 2). It was associated with fun and enjoyment and ‘is something to be enjoyed with others’ [19]; ‘Alcohol appeared to serve an important role for the majority of participants in enhancing positive situations and facilitating enjoyment and socialising with friends and acquaintances’ [19]; ‘Life would be less enjoyable without it’ [15].

It was often used at special occasions or as a treat such as with a good quality wine [19], or to bring something special to the day, for example, ‘a regular evening is enhanced by a glass of wine or as a prize after completing some work’ [20]. Social drinking was often viewed as ‘about spending time together in a cosy and relaxed atmosphere, and did not require vast amounts of alcohol’ [20].

#### Social environment, setting and social norms

People tended to adopt or share drinking habits of their partner, family members or peers [10, 14, 16, 20]. Drinking was often identified as a habit or as part of a regular routine such as a drink in the evening [10, 14, 19]. ‘Alcohol was also used as a marker to separate ‘work time’ from ‘leisure time’ or other habitual times to drink, e.g. when cooking a meal’ [19], or related to other ‘rituals, moods and environments’ [20]. One study linked alcohol use in older people to ‘maintaining routines and identity in the context of role loss and functional limitations’ [14].

There was evidence that older people often have their own views about socially acceptable drinking and disapprove of excessive drinking in older people [9, 15, 19]. ‘Some of the subjects talked disapprovingly of older people or their peers whose drinking habits did not live up to the ideal of moderation’ [20]. This was sometimes presented in moralistic terms and there was stigma associated with problem drinking, often linked to attitudes they were brought up with as children, such as the temperance movement in their youth [9, 15, 22]. There was also some evidence that older people continue into older age the drinking patterns they develop earlier in the lifecourse [9, 14, 15].

Those living independently in retirement villages reported the availability of social groups in that setting which often involved alcohol [14, 19] with drinking sometimes associated with a sense of continuity ‘having a drink in these settings may signify that life is going forwards’ [14]. ‘Drinking could depend on the type of social activity and who else was present’ [19] and social pressures could apply in communal residential settings where drunkenness was frowned upon [19]. Some social environments were associated with heavy drinking such as predominantly male environments related to sport [11].

Evidence relating to drinking around the retirement transition was mixed [9, 14, 15, 19, 20]. In some studies, participants noted that reduced opportunities for socialisation since retiring had reduced their alcohol consumption [19, 20] others suggested their alcohol use had increased because of more opportunities for socialisation [19, 20] or ‘they were freed from daily responsibilities of work and family and had more disposable income that enabled them to enjoy social drinking’ [15].

#### Health-related issues

Drinking alcohol for medicinal purposes or for health benefits was often cited, such as for heart disease or to relieve physical symptoms [9, 18, 22]. As Tolvanen 2005 noted, ‘appealing to doctor’s orders is one way in which the narrators justified moderate consumption’. Aira 2008 noted that some people who did not formally report alcohol use did cite use for medicinal purposes. Using alcohol for relaxation was also reported in several studies [9, 13–15, 19, 20].

In a number of studies (Table 2) some people reported that they limited their alcohol use in older age because they couldn’t drink as much as they used to without consequences for their body. For example: ‘body can’t take it any more’ [20] or because the effects of alcohol were stronger in older age [21].

Ill health was also cited as a reason for changing drinking behaviour, such as having experienced a heart attack, stroke, other chronic conditions, use of medicines for these conditions or falls were all cited [9,15]. However, ‘continued heavy drinking was also presented as normal behaviour for someone experiencing relative wellbeing in later life, or if ill health was construed as unrelated to alcohol consumption’ [9]. Several studies highlighted scepticism about the potential health risks and medical advice about alcohol among older people: ‘there was some refusal to acknowledge health risks’ [19]; ‘some people discounted health risks associated with alcohol and medication or between alcohol intake and poor health’ [9]; ‘health was not viewed as a major facilitating or constraining factor for alcohol consumption’ [18]; ‘some older people trivialised the use and risks of alcohol’ [21].

There was confusion about health guidelines and messages relating to drinking [10] with some uncertainty about ‘units’ among older people [10]. ‘Health messages could be perceived as class-based preaching’ [10] or a perception that campaigns against excessive drinking didn’t apply to them, they targeted ‘those who got themselves drunk or lost self-control’ [10].

#### Drinking to deal with difficulties in life

Drinking to deal with negative issues was raised in several studies. Examples were: dealing with anxiety, sadness or stress, such as loneliness, partner illness, the end of a relationship, bereavement, loss of partners, family or friends, or loss of physical health and mobility or of daily routines and structures [9, 14, 15, 19–21]. These were related to periods of heavier alcohol use or less controlled drinking, and drinking secretively or alone [9]. It could also be used to mask declining abilities such as forgetfulness or reduced physical capability [11]. In some cases, solitary drinking was viewed as a sign of problem drinking [13] but drinking alone was not always negative, in the context of unwinding with a glass of wine, for example [20].

#### Access issues

Cost and availability could influence drinking behaviour in older people, in contrasting ways. Being on a low income could restrict social use of alcohol [15] or occasionally drinking more because of special offers and cheap drinks was discussed [14,15]. However, cost was sometimes not seen as a barrier due to the perception of relatively low prices by some older populations [15]**.** Korean immigrants to Canada tended to drink more moderately because of higher alcohol prices in Canada [13]. Increasing availability was also linked to higher consumption [14, 20].

#### Drinking identities

The promotion of positive alcohol identities in older people was identified and suggested as a facilitator for moderate alcohol consumption in the Wilson 2013 study [9]: ‘In positive terms, alcohol was treated as something that could be enjoyed by older people who consumed it minimally or occasionally. Alcohol was drunk through choice, and therefore implied control over one’s actions’. Similar ideas emerged in other studies, such as views of controlled use versus non-controlled use ‘self-controlled use was associated with social norms and conventions or not to disturb others’ [20]. On a similar theme, the Haarni 2010 [20] study reported that many older people have an ideal and goal of moderation but also experience of coping wisely with alcohol and Ward 2011 concluded: ‘There may be benefits in emphasising older peoples’ life experience to use alcohol wisely’ [15]. These identities were ‘defined primarily by self-control and propriety, rather than health considerations’ [9]. However, the distinction between heavy but controlled use and uncontrolled heavy use were not always clear [9].

#### Self-regulating strategies

Some older people imposed self-regulating strategies such as not drinking during the day to limit consumption [19], controlling use by moving from spirits to milder beverages [20], only drinking alcohol with meals [19], not drinking alone [13, 18], waiting till after a certain time in the day to have a drink.

#### Sources of help

GPs were identified as the primary source of help for alcohol problems in some studies [10,15] with some evidence that people might cut down drinking in response to GP advice [10,15].

#### Issues relating to specific population groups

Gender: Attitudes to drinking or to drinkers could differ by gender, although one study concluded that drinking patterns were not strongly defined by gender [9]. For example: ‘male drinkers were described as likeable characters, females who drank were perceived of as not fulfilling their duties as wives and mothers’ [22]; ‘men’s heavy consumption of beer was valorised.....while women drinking beer or going to pubs, particularly alone would be viewed negatively’ [9]; ‘heavy drinking especially among women was viewed unfavourably and considered inappropriate’ [20]. One study reported that deviations from male stereotypes such as drinking half pints instead of pints could be a barrier to attempts to reduce alcohol consumption [9].

Cultural: In some immigrant populations, alcohol use and type was related to recreation of their homeland cultures and national identities [11, 13].

Older people that received homecare services: In a UK (Scotland) study of the views of carers and managers providing homecare services to elderly people [12], homecare workers were wary about reporting alcohol problems in their older clients, as they were concerned they would stop trusting them; older people often received free delivery for bulk orders, so would purchase more alcohol than necessary.

## Discussion

This systematic review collates and synthesises evidence from 14 qualitative studies relating to older people and alcohol use.

### Summary of key findings and interpretation

Alcohol use is strongly associated with social engagement and enjoyment in older people across studies from different countries and cultures. There is scepticism about the health risks of alcohol and medical advice and older people can sometimes be unclear about what is excessive drinking. It is often associated with their regular routines and can be an integral part of life, relating to identity and continuity. However, older people are a heterogeneous population and conversely, drinking was also linked to difficulties such as social isolation, stress, illness or bereavement. Older people often employ strategies to control their own alcohol intake. Alcohol messages and interventions in older people could emphasise the accumulated life experience of older people to drink wisely.

Potential alcohol reduction and prevention strategies in older people developed from the evidence in this review, that may be beneficial, are summarised in Fig 2. The evidence supports a strong social role for drinking alcohol which must be taken into account in any policy development with the potential benefits of social and community participation for cognitive health. Approaches to reducing alcohol harm need to avoid paradoxical harm with a need for cultural approaches that reduce the damage of drinking alcohol but retain the benefit of socialising.

### Scope and limitations

Most of the studies identified by this review were conducted in people drinking at a range of consumption levels. In ‘at-risk’ drinkers, different issues might be identified or prioritised. Most intervention studies to date have targeted at-risk drinkers so more specific information about context, barriers and facilitators in at-risk older drinkers is needed to inform future intervention design. Only one mixed-methods study with very limited qualitative data was conducted with solely ‘at-risk’ drinkers [17]. In that study, participants who made no changes to their drinking after an alcohol reduction intervention included habit, social life, and enjoyment as barriers to change and other factors which were consistent with the overall findings of this review, but the limited data reported was insufficient to make robust conclusions [17]. It has also been reported that ‘at risk’ older drinkers may be younger (younger-old) [23] and that a higher proportion of ‘at-risk’ users drink to deal with difficulties, such as, ‘meaningless life’ ‘relieving anxiety’, ‘relieving loneliness’ and ‘relieving depression’ [23].

The older population included in the review was people aged 55 and over. This relatively low age cut-off was chosen to reflect potential for earlier disease onset relating to inequalities. Most qualitative studies had fewer than 50 participants. The two studies with very limited qualitative components were mixed methods studies that also collected quantitative data and had larger sample sizes.

Few studies reported socioeconomic status of participants and of those, none were conducted specifically in low SES groups. It is well-reported that those of low SES are more difficult to engage in research studies [24]. However, alcohol-related harm is higher in socioeconomically disadvantaged populations [25]. It is possible that participants recruited to these studies were those older people who are most socially active anyway so may not reflect the experiences of those who may be more isolated. Future qualitative studies could target those of low SES, those who are less socially engaged or focus more on heavy or ‘at-risk’ drinkers.

Searches were limited to studies in English and OECD countries from 2000 onwards so there is a risk that other relevant studies were not identified. A cut-off date from 2000 was chosen because of the changing characteristics of older people over recent years. One study was excluded as it was specifically in people with chronic health conditions [26], however, it reiterates many of the issues identified in this review [26]. In the studies included in this review, the health status of participants was not formally reported but it is likely that participants included both healthy older people and those with chronic health conditions.

Since the searches were conducted for this review, we are aware of one study [27] published after the search cut-off date that would be eligible for inclusion in this review. This paper also further reinforces many of the findings of the current review. For example: ‘The strategies employed by older people to curb their drinking can be promoted and encouraged in public health messages as a way of reducing overall alcohol use, without stigmatising older people’s drinking’ [27].

### Relationship of evidence to previous interventions

A recent systematic review of interventions to prevent or reduce excessive alcohol consumption in older people identified 13 studies, most relating to reduction of drinking in ‘at risk’ drinkers, with few primary prevention studies in older people [28]. That review found some evidence that more intensive interventions involving personalised feedback, physician advice and educational materials with follow-up may be most effective in older people. The current review reinforces that physician advice may be helpful in prompting people to cut down their drinking. Personalised feedback may also help older people identify when their drinking is excessive. Extended follow-up may provide some social support and routine, that drinking currently provides. However, to date, alcohol reduction and prevention interventions have not addressed the importance of alcohol to social life and the consequences of reducing drinking to social and societal participation. Additionally, many of the other contextual factors raised by this review have not been addressed in intervention design. There is scope to design future interventions building on the potential strategies generated by this review and consideration of context.

Recent discussions in the literature have highlighted the difficulties of translating positive results of alcohol reduction interventions from research into practice [29]. This can be because those recruited to research trials are often motivated to reduce drinking. However, the current review demonstrates how closely linked alcohol can be to social life and routines, and strategies to reduce alcohol consumption may need to provide alternatives to these factors that are fundamental to the structure of life, while also considering the health impact of drinking. As stated by Ward et al [15]: ‘There is a need to understand the context in which drinking is occurring, the role it plays in older people’s lives and what might be the consequences of stopping drinking’.

A summary of the work presented in this paper has previously contributed to an evidence-based resource for local authority commissioners, clinical commissioning groups and providers of lifestyle behaviour change programmes of interventions to help the uptake and maintenance of healthy behaviours and promote cognitive health among older adults living in the community [30]. This paper updates that evidence and provides further details of the methods, analysis and synthesis to inform the development of context sensitive interventions and policies to mitigate alcohol-related harms in older adults.

## Supporting information

S1 File Search strategies and grey literature sources. (PDF)

S2 File Excluded studies and reason for exclusion. (PDF)

S3 File Further characteristics of included studies. (PDF)

S4 File Quality assessment for qualitative studies of alcohol in older people. (PDF)

S5 File PRISMA checklist. (DOCX)
