# Supplementary material for: Alcohol and older people: A systematic review of barriers, facilitators and context of drinking in older people and implications for intervention design
Source: PLoS One. 2018 Jan 25;13(1):e0191189. doi: 10.1371/journal.pone.0191189 (PMC5784942; doi:10.1371/journal.pone.0191189)
Supplement: S1 File — (PDF) [file pone.0191189.s001.pdf]

Supplement 1. Kelly et al, Alcohol and older people: a systematic review of barriers, facilitators and context of drinking in older people and implications for intervention design.

Search strategies and grey literature sources

The alcohol searches were part of broader searches for a series of reviews covering a range of health behaviours. Searches were conducted for 1) any existing systematic reviews 2) primary studies (primary qualitative studies and primary intervention studies – findings from intervention studies have been reported separately). Original searches were conducted in July-Nov 2015 and updated to end February 2017. A search filter was used to identify qualitative studies (The InterTASC Information Specialists' Sub-Group Search Filter Resource (ISSG) available at:- https://sites.google.com/a/york.ac.uk/issg-search-filters-resource/). For qualitative studies, searches were conducted using: the University of Texas School of Public Health filter; for systematic reviews the SIGN filter was used.

1. Search dates

**Database (source)**	**Search dates (systematic reviews)**	**Search dates (primary intervention and qualitative studies)**
MEDLINE (Ovid SP)	28-2-2017	28-2-2017
EMBASE (Ovid SP)	28-2-2017	28-2-2017
PsycINFO (EBSCO host)	28-2-2017	28-2-2017
CINAHL (Cumulative Index to Nursing and Allied Health Literature) EBSCO host	28-2-2017	28-2-2017
CENTRAL (Cochrane Central Register of Controlled Trials) http://www.cochranelibrary.com/about/central-landing-page.html	28-2-2017	28-2-2017
Social Sciences Citation Index (Web of Science)	28-2-2017	28-2-2017
Cochrane Library http://www.cochranelibrary.com/		N/A
York Centre for Reviews and Dissemination Health Technology Assessment (HTA); NHS Economic Evaluation Database (NHS-EED); Database of Abstracts of Reviews of Effectiveness (DARE) (Note: NHS-EED and DARE records only available up to 31-3-2015)	28-2-2017	N/A
Grey literature (searched for SRs and primary studies)	21-02-2017	

2. Example search strategy for MEDLINE (for a range of health behaviours including alcohol)

1. exp Health Behavior/
2. exp Risk Reduction Behavior/
3. exp Health Promotion/
4. exp Primary Prevention/
5. exp Preventive Medicine/
6. ((health$ adj3 behavior$) or behaviour$).ab,ti.

7. ((behavio?r$ or lifestyle or "lifestyle") adj3 (change$ or changing or modification or modify or modifying or therapy or therapies or program$ or intervention$ or counsel$)).ab,ti.
8. ((ageing or aging) adj3 (well or success$ or positive$ or active$ or healthy)).ab,ti.
9. ("health check" or "check up" or "check-up").ab,ti.
10. "health MOT".ab,ti.
11. "NHS check".ab,ti.
12. or/1-11
13. exp Diet/
14. *Food/
15. (diet or diets or dietary).ab,ti.
16. (dietary adj3 fat$).ab,ti.
17. salt$.ab,ti.
18. sugar$.ab,ti.
19. fruit$.ab,ti.
20. vegetable$.ab,ti.
21. (wholegrain or whole-grain or "whole grain" or "glyc?emic index").ab,ti.
22. (fish or "omega-3" or "omega-6" or "omega 3" or "omega 6" or (fish adj2 oil$)).ab,ti.
23. ("five a day" or "5 a day").ab,ti.
24. (fat$ adj3 (intake$ or diet$ or consum$)).ab,ti.
25. ((protein or carbohydrate$ or fibre or fiber) adj2 (intake$ or consum$ or diet$)).ab,ti.
26. ((energy or calorie$) adj2 (intake$ or consum$ or diet$)).ab,ti.
27. (vitamin$ adj3 (intake$ or consum$ or diet$)).ab,ti.
28. ((micronutrient or micro-nutrient or "micro nutrient") adj3 (intake$ or consum$ or diet$)).ab,ti.
29. nutrition.ab,ti.
30. *Food Habits/
31. *Food Preferences/
32. exp Nutrition Therapy/
33. or/13-32
34. eat$.ab,ti.
35. (over eat or "over eat" or overeat).ab,ti.
36. *Malnutrition/
37. malnutrition.ab,ti.
38. (undernutrition$ or undernourish$ or under-nutrition$ or under-nourish$).ab,ti.
39. (weight adj2 (gain$ or loss$ or cycling or reduc$ or maint$ or decrease$ or increas$ or watch$ or control$ or change$)).ab,ti.
40. ((bmi or "body mass index") adj2 (gain$ or los$ or cycling or reduc$ or maint$ or decrease$ or increas$ or watch$ or control$ or changes$)).ab,ti.
41. (obesity adj2 "related behaviour").ab,ti.
42. or/34-41
43. exp Exercise/

44. exp Sports/
45. *Exercise Therapy/
46. exp Physical Exertion/
47. exp "Physical Education and Training"/ or exp Physical Fitness/
48. exp running/ or exp swimming/ or exp walking/
49. exp Bicycling/
50. exp Dancing/
51. "tai chi".ab,ti.
52. tai ji/ or yoga/
53. "tai ji".ab,ti.
54. yoga.ab,ti.
55. ((center$ or centre$ or program$ or site$ or setting$ or venue$ or event$) adj3 (sport$ or exercise$ or fitness or training$ or activ$)).ab,ti.
56. exp Life Style/
57. exp Sedentary Lifestyle/
58. sedentary.ab,ti.
59. (exercis$ or sport$ or danc$ or run$ or walk$ or jog$ or garden$ or leisure or recreation$ or golf$ or tennis$ or badminton$ or bowl$ or curl$).ab,ti.
60. (bicycl$ or training or trainer$ or bik$ or wellness).ab,ti.
61. balanc$.ab,ti.
62. ((resistance or conditioning) adj2 training).ab,ti.
63. ((cardio$ or aerobic$) adj2 (sport$ or exercise$ or fitness or training$ or activ$)).ab,ti.
64. or/43-63
65. Smoking/
66. exp Smoking Cessation/
67. exp "Tobacco Use Cessation Products"/
68. (smok$ adj3 (cessation or cease$ or quit$ or stop$ or reduce$ or reduction)).ab,ti.
69. or/65-67
70. exp Drinking Behavior/
71. exp Alcohol Deterrents/
72. exp Temperance/
73. ((alcohol or drunk$ or drink$) adj3 (consum$ or misus$ or abuse$ or intoxicat$ or harmful or excess$ or binge$ or hazardous$ or heavy or temperance or abstinence)).ab,ti.
74. (temperan$ or teetotal$).ab,ti.
75. or/70-74
76. (cognit$ adj2 stimulat$).ab,ti.
77. cognit$ exercis$.ab,ti.
78. (cognit$ adj2 (stimulat$ or train$ or exercis$)).ab,ti.
79. (brain adj2 (stimulat$ or train$ or exercis$)).ab,ti.
80. puzzle$.ab,ti.

81. crossword$.ab,ti.

82. reading.ab,ti.

83. (intellect$ adj2 activit$).ab,ti.

84. or/76-83

85. (socialis$ or socializ$).ab,ti.

86. (social$ adj2 (activit$ or stimulat$)).ab,ti.

87. (social adj3 (isolat$ or network$ or contact$ or alienat$)).ab,ti.

88. lonel$.ab,ti.

89. exp Loneliness/

90. or/85-89

91. 12 or 33 or 42 or 64 or 69 or 75 or 84 or 90

92. vision tests/

93. Eyeglasses/

94. vision disorders/

95. computer terminals/

96. Asthenopia/

97. ((sight or eyesight or vision or eye$) adj2 (protect$ or maintain$ or maintenance)).ti,ab.

98. or/92-97

99. hearing tests/

100. hearing loss/

101. hearing aids/

102. ((hearing or noise$) adj2 (protect$ or maintain$ or maintenance)).ti,ab.

103. or/99-102

104. 91 or 98 or 103

105. "sun burn".ab,ti.

106. Sunburn/

107. Sunscreening agents/

108. (sun adj (light or exposure or overexposure or screen* or protect* or tan$)).ab,ti.

109. vitamin D.ab,ti.

110. Vitamin D/

111. or/105-110

112. 104 or 111

113. *Aged/

114. Retirement/

115. *Aging/

116. Geriatrics/

117. older.ab,ti.

118. elder$.ab,ti.

119. senior$.ab,ti.

120. geriatr$.ab,ti.

121. retir$.ab,ti.

122. ag?ing.ab,ti.

123. longevity.ab,ti.

124. "later life".ab,ti.

125. "randomized controlled trial".pt.

126. (random$ or placebo$ or "single blind$" or "double blind$" or triple blind$).ab,ti.

127. (retraction of publication or retracted publication).pt.

128. ((comment or editorial or meta-analysis or practice-guideline or review or letter or journal correspondence) not "randomized controlled trial").pt.

129. (random sampl$ or random digit$ or random effect$ or random survey$ or random regression).ab,ti.

130. controlled trial.pt.

131. 129 not 130

132. 125 or 126 or 127

133. 128 or 131

134. 132 not 133

135. or/113-124

136. 112 and 134 and 135

137. limit 136 to yr="2000 -Current"

138. Animals/

139. Humans/

140. 138 and 139

141. 138 not 140

142. 137 not 141

143. limit 142 to english language

3. For qualitative studies searches were as above to line 124 then the following filter for qualitative studies was used (University of Texas School of Public Health filter, The InterTASC Information Specialists' Sub-Group Search Filter Resource (ISSG) available at:- https://sites.google.com/a/york.ac.uk/issg-search-filters-resource/):- "semi-structured" or semistructured or unstructured or informal or "in-depth" or indepth or "face-to-face" or structured or guide) adj3 (interview* or discussion* or questionnaire*))).ti,ab. or (focus group* or qualitative or ethnograph* or fieldwork or "field work" or "key informant").ti,ab. or interviews as topic/ or focus groups/ or narration/ or qualitative research/

4. For non-randomised studies, searches were as above to line 124 then the following filter for non-randomised studies was used:-

evaluation studies/ or evaluation studies as topic/ or program evaluation/ or validation studies as topic/ or ((pre- adj5 post-) or (pretest adj5 posttest) or (program* adj6 evaluat*)).ti,ab. or (effectiveness or intervention).ti,ab.

Supplement 1. Kelly et al, Alcohol and older people: a systematic review of barriers, facilitators and context of drinking in older people and implications for intervention design.

5. Grey literature sources searched and dates

Sites	Date	Refs	Date	Refs	TOTAL
Action on Hearing Loss	06.10.14	2	21.02.17	2	4
Alzheimer's Society	06.10.14	6	21.02.17	22	28
Beth Johnson Foundation	06.10.14	7	21.02.17	1	8
British Library	06.10.14	130	21.02.17	25	155
Campbell Collaboration	08.10.14	4	21.02.17	0	4
Cochrane	07.10.14	68	21.02.17	3	71
Department of Health	07.10.14	9	21.02.17	0	9
E-Print Network	21.10.14	42	21.02.17	3	45
Fight For Sight	07.10.14	0	21.02.17	0	0
Google Scholar	07.10.14, 08.10.14	360	21.02.17	52	412
Grey Literature Report	20.10.14	49	21.02.17	27	76
Health Evidence Canada	21.10.14	112	21.02.17	23	135
Lenus	21.10.14	58	21.02.17	17	75
NHS Evidence	21.10.14 22.10.14	208	21.02.17	14	232
NYAM	22.10.14	31	21.02.17	3	34
OAister	22.10.14	116	21.02.17	10	126
Open Grey	22.10.14, 27.10.14	67	21.02.17	0	67
Public Health Obs	27.10.14	12	21.02.17	0	12
Public Health Europe	27.10.14	44	21.02.17	2	46
RAND	27.10.14	159	21.02.17	5	164
RNIB	27.10.14	14	21.02.17	0	14
Science Direct	27.10.14, 28.10.14	156	21.02.17	42	198
WHO	28.10.14	26	21.02.17	4	30
