# Supplementary material for: Alcohol and older people: A systematic review of barriers, facilitators and context of drinking in older people and implications for intervention design
Source: PLoS One. 2018 Jan 25;13(1):e0191189. doi: 10.1371/journal.pone.0191189 (PMC5784942; doi:10.1371/journal.pone.0191189)
Supplement: S2 File — (PDF) [file pone.0191189.s002.pdf]

Supplement 2. Kelly et al, Alcohol and older people: a systematic review of barriers, facilitators and context of drinking in older people and implications for intervention design.

Excluded studies and reason for exclusion

Only excluded studies relevant to alcohol reported below: searches included a range of health behaviours.

Study	Reason excluded
Nicholson D, McCormack F, Seaman P, Bell K, Duffy T, Gilhooly M: Alcohol and healthy ageing: a challenge for alcohol policy. *Public Health* 2017, 148:13-18.	Meets inclusion criteria but published after search cut off dates. Has been discussed in the discussion section of the review
**Gavens L, Goyder E, Hock ES, Harris J, Meier PS. Alcohol consumption after health deterioration in older adults: a mixed-methods study. *Public Health* 2016; 139:79–87.**	**Has a qualitative component of relevance to B/F but only recruited people with existing major health conditions (excluded from this review), who may have reduced drinking in response to illness**
Pettigrew S, Pescud M: The salience of alcohol-related issues across the adult lifespan. *Health Education Journal* 2016, 75(1):117-128.	Only included people aged 18-65, excluded people aged 65+. Sub group of older people comprised people aged 46 to 64 so results only relevant to 46 to 64 age group, mean age of that population lower than the cut-off age for inclusion (55+ yrs)
Immonen S, Valvanne J, Pitkala KH: Older adults' own reasoning for their alcohol consumption. *Int J Geriatr Psychiatry* 2011, 26(11):1169-1176.	Not formally a qualitative study – uses pre-defined responses. Has a very small section asking why people made changes to their drinking but very limited data
Bakhshi S, While AE: Older people and alcohol use. *Gastrointestinal Nursing* 2015, 13(Sup10):S27-S32.	Review, not SR, not B/F
Bamberger PA: Winding Down and Boozing Up: The Complex Link Between Retirement and Alcohol Misuse. *Work Aging and Retirement* 2015, 1(1):92-111.	Review, not SR, not focused on qualitative studies
Barry KL, Blow FC: Drinking Over the Lifespan: Focus on Older Adults. *Alcohol Research: Current Reviews* 2016, 38(1):115-120.	Review, not SR, not B/F
Britton A, Bell S: Reasons why people change their alcohol consumption in later life: Findings from the Whitehall II Cohort Study. *PLoS ONE* 2015, 10 (3) (no pagination) (e0119421).	Not qualitative study
Collum SP: The effect of social support and quality of life on attitudes towards drinking in a population of older adults. US: ProQuest Information & Learning; 2015.	Not qualitative study

Supplement 2. Kelly et al, Alcohol and older people: a systematic review of barriers, facilitators and context of drinking in older people and implications for intervention design.

Draper B: Barriers and facilitators to accessing specialist drug and alcohol services for older people with risky substance use. *International Psychogeriatrics* 2015, 27:S25-S26.	Potentially relevant but abstract only. Checked for full paper but none found.
Enggasser JL, Hermos JA, Rubin A, et al. Drinking goal choice and outcomes in a Web-based alcohol intervention: results from VetChange. *Addictive Behaviors* 2015; 42:63–8.	Not older people - Mean age 31-33
Fitzgerald N, Platt L, Heywood S, McCambridge J: Large-scale implementation of alcohol brief interventions in new settings in Scotland: a qualitative interview study of a national programme. *BMC Public Health* 2015, 15:289.	No data specific to older people
Khan N, Wilkinson J, Keeling S: Reasons for changing alcohol use among older people in New Zealand. *Australasian Journal on Ageing* 2006, 25(2):97.	Used a questionnaire to collect data with pre-defined questions which were checked against open-ended responses so not really qualitative data.
Dent OF, Grayson DA, Waite LM, Cullen JS, Creasey H, Broe GA: Alcohol consumption in a community sample of older people. *Australian and New Zealand journal of public health* 2000, 24(3):323-326.	Not qualitative study
Dias P, Oliveira A, Lopes C: Social and behavioural determinants of alcohol consumption. *Annals of Human Biology* 2011, 38(3):337-344.	Not qualitative study, not older people
Hajat S, Haines A, Bulpitt C, Fletcher A: Patterns and determinants of alcohol consumption in people aged 75 years and older: Results from the MRC trial of assessment and management of older people in the community. *Age and Ageing* 2004, 33(2):170-177.	Not qualitative study
McDougall Jr GJ, Becker H, Delville CL, Vaughan PW, Acee TW: Alcohol use and older adults: A little goes a long way. *International Journal on Disability and Human Development* 2007, 6(4):431-440.	Not qualitative study
Molander RC, Yonker JA, Krahn DD: Age-related changes in drinking patterns from mid- to older age: results from the Wisconsin longitudinal study. *Alcoholism: Clinical & Experimental Research* 2010, 34(7):1182-1192.	Not qualitative study
Nuevo R, Chatterji S, Verdes E, Naidoo N, Ayuso-Mateos JL, Miret M: Prevalence of alcohol consumption and pattern of use among the elderly in the WHO European region. *European Addiction Research* 2015, 21(2):88-96.	Not qualitative study
Tredal I, Soares JF, Sundin Ö, Viitasara E, Melchiorre MG, Torres-Gonzales F, Stankunas M, Lindert J, Ioannidi-Kapolou E, Barros H: Alcohol use among abused and non-abused older persons aged 60–84 years: An European study. *Drugs: Education, Prevention & Policy* 2013, 20(2):96.	Not qualitative study
