# Supplementary material for: Alcohol and older people: A systematic review of barriers, facilitators and context of drinking in older people and implications for intervention design
Source: PLoS One. 2018 Jan 25;13(1):e0191189. doi: 10.1371/journal.pone.0191189 (PMC5784942; doi:10.1371/journal.pone.0191189)
Supplement: S3 File — (PDF) [file pone.0191189.s003.pdf]

Supplement 3. Kelly et al, Alcohol and older people: a systematic review of barriers, facilitators and context of drinking in older people and implications for intervention design.

Further characteristics of included studies

Study	Study design	Country	Age (years)	Population and setting	Study objective	Overall summary of study quality ± (for qualitative components)
**Qualitative studies**						
Burrus 2015	In-depth interviews (n=11)<br><br>(part of a mixed methods study)	US	Mean: 81.5 (7.5); Range 68-90	N=11 older adults living independently in a congregate retirement community, who were regular drinkers. The setting included onsite pubs and retail shops offering alcohol<br><br>Gender: 45.5% male; 54.5% female<br>SES: Not reported<br>Ethnicity: 100% Caucasian<br>Alcohol consumption: Regular drinkers only included (drank on at least 6 of 8 days assessed). Participants drank on 92% of days in the last 8 days; with mean of 1.88 drinks on days people drank	Understanding older adults attitudes and beliefs about drinking: perspectives of residents in congregate living	+
Dare 2014	Qualitative study (in-depth interviews)	Australia	65-74<br><br>(Mean: 69.7 (SD 3.3))	N=20 men and N=22 women who were living in either private residences or (secular, resident-funded) retirement villages<br><br>Gender: 47.6% male; 52.4% female<br><br>SES: Participants from areas classified as having higher levels of socioeconomic advantage and >50% had a post-school qualification.<br><br>Ethnicity: not reported	To identify relationships between social engagement, setting and alcohol use	++

Study	Study design	Country	Age (years)	Population and setting	Study objective	Overall summary of study quality ± (for qualitative components)
				Alcohol consumption: Over 75% drinking alcohol more than 4 days/wk. Average daily consumption of alcohol based on quantity/frequency (standard drinks): Private home; men 1.89 (1.4); women 1.21 (0.8); Retirement village: men 3.13 (4.4); women 1.68 (1.1)<br><br>The majority of men and women were drinking at low risk classification levels based on WHO at-risk drinking classification (based on 2000 criteria)		
Haarni 2010<br><br>(Too Much is Always Too Much – Alcohol and Ageing project)	Qualitative (interviews)	Finland	60-75	N=31 Urban older adults<br><br>Gender: 48.4% male; 51.6% female<br><br>SES: 14 had passed the matriculation examination, eight the middle school, and eight had at least the equivalent of elementary school studies. 28 were retired, 1 unemployed, 2 still working; 'Prior to retirement…had worked in a variety of blue and white collar professions in occupations ranging from managing director to auxiliary nurse'<br><br>Ethnicity: not reported<br><br>Alcohol consumption: Current or ex-consumers of alcohol were included. People who had abstained from alcohol all their life were excluded. Reports 'the study included many kinds of alcohol consumers' –	Life experience and alcohol: 60-75 year olds relationship to alcohol	-

Study	Study design	Country	Age (years)	Population and setting	Study objective	Overall summary of study quality ± (for qualitative components)
				included range of drinkers, including large, average and small amounts and frequencies		
Haighton 2016<br><br>(same sample as Wilson 2013)	Qualitative (Interviews (N=24) and focus groups (N=27 people)	UK (North East England)	50+<br>Range: 50-95	N=24 (qualitative interviews) and N=27 (focus groups) older adults recruited through Age UK and regional services for alcohol problems who had experience of drinking alcohol at any level of consumption<br>Gender: 50% male; 50% female (interviews); 22.2% male; 77.8% female<br>SES: Not reported<br>Ethnicity: not reported<br>Alcohol consumption: Those recruited had a range of patterns of consumption including occasional minimal drinkers, moderate and heavy drinkers, previously dependent drinkers (including those now abstinent and those currently drinking at sensible levels) and 2 currently dependent drinkers	Experiences of and attitudes towards services providing support for alcohol related health issues in people aged 50 and over	++
Johannessen 2015	Qualitative (interviews)	Norway	65+<br><br>(range 65 to 92; mean: 81)	N=16 older people that received in-home nursing service or home-help services (N=14 were widows or widowers)<br><br>Gender: 37.5% male; 62.5% female<br><br>SES: Not reported<br><br>Ethnicity: not reported<br><br>Alcohol consumption: 15 (of 16) had used alcohol.	Older peoples' experience with and reflections on use and misuse of alcohol and psychotropic drugs	++

Supplement 3. Kelly et al, Alcohol and older people: a systematic review of barriers, facilitators and context of drinking in older people and implications for intervention design.

Study	Study design	Country	Age (years)	Population and setting	Study objective	Overall summary of study quality ± (for qualitative components)
Joseph 2012	Qualitative (in-depth formal interviews, observation, casual conversation, informal interviews)	Canada	44 to 74 (Mean: 61)	Older male cricket players of Afro-Caribbean origin (friendly, non-league) and spectators (male and female). N=27 formal interviews plus data collected by observation, casual conversation.<br><br>Gender: Predominantly male<br><br>SES: Not reported<br><br>Ethnicity: Afro-Caribbean<br><br>Alcohol consumption: Most of the participants appeared to drink heavily but not specifically reported	Alcohol and older Caribbean-Canadian men	+
Kim 2009	Qualitative (focus group)	Canada	60+<br><br>62 to 83 (range); mean age 72 (SD 5.94)	N=19 elderly Korean immigrants residing in Canada (14 men, 5 women)<br><br>Gender: 26.3% male; 73.7% female<br><br>SES: Not reported<br><br>Ethnicity: Korean<br><br>Alcohol consumption: Drinking alcohol was a criterion for recruitment. 63.2% drank more than once a week	To explore drinking culture, alcohol and alcohol use in older Korean immigrants in Canada	+
Millard 2008	Qualitative	UK	65+	N= 90 staff and managers providing home, day, and	Alcohol and service	-

Study	Study design	Country	Age (years)	Population and setting	Study objective	Overall summary of study quality ± (for qualitative components)
	(focus groups)	(Scotland)		residential care to elderly clients.<br><br>Gender: Not reported for staff/managers or elderly clients<br><br>SES: Not reported for staff/managers or elderly clients<br><br>Ethnicity: not reported<br><br>Alcohol consumption: Not reported for staff/managers or elderly clients	gaps in homecare for older people: including how client's alcohol problems were identified, role of home care provider, barriers to seeking help	
Reczek 2016	In-depth interviews (n=88); interviews conducted between 2003 and 2009	US	1) Mean: 63.5; Range 40-87<br>2) Mean 59; Range 40-89	1) Participants were both spouses in 21 long-term (>7 years) heterosexual marriages (n=42); average marital duration was 32 years; 2 of the 21 currently married women were previously widowed, 5 were previously married; 2 of the 21 currently married men were previously widowed, 4 had been previously married<br>2) A second sample (n=46) included men and women in their first marriages, those who had remarried, currently divorced, never married or widowed participants<br>Gender: 1) and 2) 50% male, 50% female<br>Ethnicity: 1) 88% white, 12% African American; 2) 50% white, 50% African American<br>SES: 1) Mean income $ 60,000; Range ($ 40,000 to $120,000): (based on 2003 to 2006 data); 2) Mean	Relationships between marital history and alcohol use in older adults	+

Supplement 3. Kelly et al, Alcohol and older people: a systematic review of barriers, facilitators and context of drinking in older people and implications for intervention design.

Study	Study design	Country	Age (years)	Population and setting	Study objective	Overall summary of study quality ± (for qualitative components)
				$52,200; Range $0-120,000 (based on 2008-2009 data)<br>Alcohol consumption: 1) and 2) Not reported		
Tolvanen 2005 Vitality 90+ project	Qualitative (interviews)	Finland	90+	N=181 participants who mainly lived in their own homes though some were in service housing or in nursing homes<br><br>Gender: 33.5% male; 76.5% female (of interviews that discussed alcohol)<br><br>SES: Not reported<br><br>Ethnicity: not reported<br><br>Alcohol consumption: 63% of men and 34% of women currently used alcohol; 23% of men and 6% of women had previously used alcohol earlier but no longer drank; and 13% of men and 24% of women did not drink at all (based on those who reported consumption in interviews)	Alcohol in life story interviews with Finnish people aged 90 or over	+
Ward 2011	Qualitative (N=21 interviews and N=3 focus groups with older people)	UK	Range: mid 50s to late 80s.	N=21 individual interviews and N=3 focus groups. Aimed to include a diverse range of older people. Included people living in their own homes, in sheltered housing and in hostels<br><br>Gender: 61.9% male, 29.1% female (interviews)<br><br>SES: not reported	Older people's perspectives on alcohol use in later life	+

Study	Study design	Country	Age (years)	Population and setting	Study objective	Overall summary of study quality ± (for qualitative components)
				Ethnicity: 100% white for interviews but one focus group conducted with black people (explored general views only)<br><br>Alcohol consumption: Not specifically reported but all participants appeared to consume alcohol. 'Aim was to recruit people who regularly drink alcohol for whom it may or may not be a problem'		
Wilson 2013<br><br>(same sample as Haighton 2016)	Qualitative<br><br>Interviews (N=24) and 3 focus groups (N=27 people)	UK (North East England)	50+<br>Range: 50-95	N=24 (qualitative interviews) and N=27 (focus groups) older adults recruited through Age UK and regional services for alcohol problems who had experience of drinking alcohol at any level of consumption<br><br>Gender: 50% male; 50% female (interviews); 22.2% male; 77.8% female.<br><br>SES: Not reported<br><br>Ethnicity: not reported<br><br>Alcohol consumption: Those recruited had a range of patterns of consumption including occasional minimal drinkers, moderate and heavy drinkers, previously dependent drinkers (including those now abstinent and those currently drinking at sensible levels) and 2 currently dependent drinkers	To understand older people's reasoning about drinking in later life and how this interacted with health concerns	++

Supplement 3. Kelly et al, Alcohol and older people: a systematic review of barriers, facilitators and context of drinking in older people and implications for intervention design.

Study	Study design	Country	Age (years)	Population and setting	Study objective	Overall summary of study quality ± (for qualitative components)
**Studies with a limited qualitative component**						
Aira 2008<br>(Part of the Geriatric Multidisciplinary Strategy for Good Care of the Elderly Study;GeMS)	Qualitative (interviews) and quantitative assessment of alcohol use using AUDIT-questionnaire	Finland	75+<br><br>(83.4% aged 75-84; 16.6% aged 85+)	N=699 home-dwelling elderly living in the community<br><br>Gender: 30.5% male; 69.5% female.<br><br>SES: 18.0% had >9 years education; 52.0% had 4-9 years education; 17.4% < 4 years education<br><br>Ethnicity: not reported<br><br>Alcohol consumption: 48.5% had used alcohol in past year; 51.5% had abstained in past year. 19.7% had used alcohol for medicinal purposes (63% of these reported abstaining). Similar in males and females	To describe alcohol use as self-medication by people aged over 75 years	-
Borok 2013	Telephone interviews	US	Mean: 68.7 SD 6.6	N=399 older 'at-risk' drinkers (identified by screening) who had taken part in an RCT to reduce drinking<br><br>Gender: 69% male, 31% female.<br><br>SES: 49% had college degree or more; 29% had some college; 22% high school or less<br><br>Ethnicity: 88% white, 8% Hispanic/Latino, 3% other	To understand why older at-risk drinkers decide to increase, decrease or maintain alcohol consumption after participation in an RCT aimed at reducing drinking	-

Supplement 3. Kelly et al, Alcohol and older people: a systematic review of barriers, facilitators and context of drinking in older people and implications for intervention design.

Study	Study design	Country	Age (years)	Population and setting	Study objective	Overall summary of study quality ± (for qualitative components)
				Alcohol consumption: Only at-risk users included: identified by screening with CARET		

± Overall summary of study quality using the methodology and detailed assessment of the domains shown in S4.
