# Supplementary material for: Alcohol and older people: A systematic review of barriers, facilitators and context of drinking in older people and implications for intervention design
Source: PLoS One. 2018 Jan 25;13(1):e0191189. doi: 10.1371/journal.pone.0191189 (PMC5784942; doi:10.1371/journal.pone.0191189)
Supplement: S4 File — (PDF) [file pone.0191189.s004.pdf]

Quality assessment for qualitative studies of alcohol in older people

Note: mixed methods studies were assessed in relation to qualitative evidence only

	Theoretical approach		Study design	Data collection	Trustworthiness			Analysis						Ethics	Overall
	1	2	3	4	5	6	7	8	9	10	11	12	13	14	
Burruss 2015	Appropriate	Clear	Not sure	Appropriate	Clear	Clear	Reliable	Not sure	Rich	Reliable	Convincing	Relevant	Adequate	Adequate	+
Dare 2014	Appropriate	Clear	Defensible	Appropriate	Clear	Not sure	Reliable	Rigorous	Rich	Reliable	Convincing	Relevant	Adequate	Appropriate	++
Haarni 2010	Appropriate	Clear	Not sure	Inadequately reported	Not described	Unclear	Not sure	Not sure	Not sure	Not sure	Not sure	Partially relevant	Not sure	Not reported	-
Haighton 2016	Appropriate	Clear	Defensible	Appropriate	Clear	Clear	Reliable	Rigorous	Rich	Reliable	Convincing	Relevant	Adequate	Appropriate	++
Johanessen 2015	Appropriate	Clear	Defensible	Appropriate	Clear	Clear	Reliable	Rigorous	Rich	Reliable	Convincing	Relevant	Adequate	Appropriate	++
Joseph 2012	Appropriate	Unclear	Not sure	Appropriate	Clear	Clear	Not sure	Not sure	Rich	Not sure	Not sure	Partially relevant	Adequate	Not reported	+
Kim 2009	Appropriate	Clear	Defensible	Appropriate	Clear	Clear	Not sure	Rigorous	Rich	Reliable	Not sure	Partially relevant	Not sure	Not reported	+
Millard 2008	Appropriate	Mixed	Not sure	Inadequately reported	Not described	Unclear	Not sure	Not reported	Not reported	Not reported	Not sure	Relevant	Not sure	Not reported	-
Reczek 2016	Appropriate	Clear	Defensible	Appropriate	Clear	Not sure	Not sure	Rigorous	Rich	Not sure	Convincing	Relevant	Adequate	Appropriate	+
Tolvanen 2005	Appropriate	Mixed	Defensible	Appropriate	Clear	Not sure	Not sure	Rigorous	Rich	Not sure	Convincing	Relevant	Adequate	Not reported	+
Ward 2011	Appropriate	Clear	Defensible	Appropriate	Clear	Clear	Not sure	Not reported	Not sure	Not reported	Convincing	Relevant	Adequate	Not reported	+
Wilson 2013	Appropriate	Clear	Defensible	Appropriate	Clear	Clear	Reliable	Rigorous	Rich	Reliable	Convincing	Relevant	Adequate	Appropriate	++
Aira 2008	Appropriate	Clear	Not sure	Appropriate	Unclear	Unclear	Not sure	Not reported	Poor	Not reported	Not sure	Partially relevant	Inadequate	Appropriate	-
Borok	Appropriate	Clear	Not sure	Inadequately reported	Unclear	Clear	Not sure	Not sure	Poor	Not sure	Not sure	Relevant	Not sure	Not reported	-

Key to headings: Theoretical approach 1. Is a qualitative approach appropriate? 2. Is the study clear in what it seeks to do? **Study design** 3. How defensible/rigorous is the research design/methodology? **Data collection** 4. How well was the data collection carried out? **Trustworthiness** 5. Is the role of the researcher clearly described? 6. Is the context clearly described? 7. Were the methods reliable? **Analysis** 8. Is the data analysis sufficiently rigorous? 9. Is the data 'rich' 10. Is the analysis reliable? 11. Are the findings convincing? 12. Are the findings relevant to the aim of the study? 13. Conclusions **Ethics** 14. How clear and coherent is the reporting of ethics? (National Institute for Health and Care Excellence (NICE) Methodology checklist: qualitative studies. https://www.nice.org.uk/process/pmg4/chapter/appendix-h-quality-appraisal-checklist-qualitative-studies)
